# Impact of radiation avoidance on survival and neurocognitive outcome in infant medulloblastoma

**DOI:** 10.3747/co.v16i6.435

**Published:** 2009-12

**Authors:** L. Lafay–Cousin, E. Bouffet, C. Hawkins, A. Amid, A. Huang, D.J. Mabbott

**Affiliations:** * Department of Pediatric Oncology and Bone Marrow Transplantation, Alberta Children’s Hospital, Calgary, AB; † Pediatric Brain Tumor Program, Hospital for Sick Children, Toronto, ON; ‡ Department of Pathology, Hospital for Sick Children, Toronto, ON; § Department of Psychology, Hospital for Sick Children, Toronto, ON

**Keywords:** Infants, medulloblastoma, neurocognitive outcome

## Abstract

**Purpose:**

Concerns about radiotherapy-related neurocognitive sequelae in young children have led to deferral or avoidance of radiation in contemporary treatment for this fragile group of patients. We compared survival and neurocognitive outcome in two groups of infants with medulloblastoma who received adjuvant conventional craniospinal irradiation (csi) or reduced or no radiotherapy during an era of change in the philosophy of infant medulloblastoma treatment.

**Patients and Methods:**

From 1985 to 2007, 29 patients 3 years of age or younger were diagnosed and treated with curative intent in our institution. Children treated before 1994 received adjuvant radiation with chemotherapy; subsequently, radiation was prescribed essentially for disease progression or relapse.

**Results:**

Median age at diagnosis was 24 months (range: 1–36 months); 15 patients (52%) presented with metastatic disease at diagnosis. As part of initial treatment, 8 children received adjuvant radiotherapy with chemotherapy, and 21 children received postoperative chemotherapy only. Five children treated with chemotherapy alone are in prolonged remission. The 5-year event-free and overall survivals were 35.9% ± 9.8% and 50.2% ± 9.6% respectively. Extent of resection, metastatic status, and desmoplastic histology were not found to be significant prognostic factors.

On serial neurocognitive evaluations, patients treated with chemotherapy with or without reduced radiotherapy demonstrated improvement of intellectual function over time. Patients treated with conventional csi exhibited significantly lower intelligence quotient scores and academic performance, with the exception of receptive vocabulary.

**Conclusions:**

Avoidance of conventional csi in treatment of very young children with medulloblastoma appears to be associated with a preserved neurocognitive profile. Neurocognitive evaluation should be integrated into the primary objectives of future infant protocols.

## 1. INTRODUCTION

Medulloblastoma is the most common malignant brain tumour in children. Of all medulloblastomas, 30% occur in children under 3 years of age 1, and this population is a very challenging group because of their recognized poorer outcome and, most importantly, because of their increased susceptibility to treatment-related toxicities. Infants with brain tumours are particularly vulnerable to significant intellectual impairment after cranial irradiation 2–5. Although craniospinal irradiation (csi) improves survival, it is usually avoided in young children because of significant neurotoxicity and very adverse neurocognitive outcomes. To delay or eliminate the need for radiation at this early stage of brain maturation, a series of contemporary therapeutic studies relying on primary postoperative chemotherapy have been developed for infants and young children 6–10. Preserved intellectual outcome has been documented for survivors treated without radiation, but comprehensive long-term longitudinal data remain limited to date 11,12. Here, we report a single-institution experience in managing infants with medulloblastoma over a 20-year period and provide comprehensive serial neurocognitive data on long-term survivors.

## 2. PATIENTS AND METHODS

At the Hospital for Sick Children in Toronto, 34 consecutive patients diagnosed with medulloblastoma before the age of 3 years were treated between April 1985 and April 2007. This retrospective review of those 34 cases was approved by the research ethics board of the institution. Clinical, therapeutic, and survival data were collected from patient files. Patient outcomes included serial neurocognitive measures and endocrine status at completion of the study.

Metastatic status according to the Chang staging system 13 and extent of resection were based on the cerebrospinal fluid (csf) analysis (when available), the surgical report, and postsurgical imaging. Extent of resection was defined as follows:

- Gross total resection (gtr)—no radiologic evidence of residual tumour
- Radical subtotal resection (rstr)—a resection of less than 100%, but of 95% or more
- Subtotal resection (str)—a resection of less than 95%, but of more than 50%
- Partial resection (pr)—a resection of less than 49%, but of 10% or more
- Biopsy—a resection of less than 10%

All tumour samples were reviewed by one neuropathologist (CH) for the purposes of the study.

Clinical management evolved over the study period. Before 1994, all patients treated with curative intent received adjuvant chemotherapy and radiotherapy. After 1994, all infants with medulloblastoma were treated according to “baby brain” protocols and strategies 7,8, those successively being Baby pog (Pediatric Oncology Group) 1 and 2, and bbsfop (Societe francaise d’oncologie pediatrique).

More recently, in an attempt to improve outcome, high-dose chemotherapy (hdc) protocols were introduced into the management of these children.

### 2.1 Neuropsychological Assessment

Neurocognitive assessments were conducted for all surviving patients. These assessments included evaluation of intelligence, receptive vocabulary, visual–motor function, memory, and academic achievement. Specifically, the test battery included either the Wechsler Intelligence Scale for Children (3rd edition) or the Wechsler Preschool and Primary Scales of Intelligence–Revised depending on the age of the child and the Peabody Picture Vocabulary Test, the Beery Visual–Motor Integration Test, the Children’s Memory Scale, and the Wide Range Achievement Test (either revised or 3rd edition).

### 2.2 Statistical Analysis

Progression-free survival (pfs) was determined from the date of diagnosis to the date of disease progression, with censoring on the date of the most recent clinical assessment. Overall survival (os) was assessed from the date of the diagnosis to the date of last follow-up or the date of death. Kaplan–Meier analysis was used to estimate os and pfs, and the log-rank test was used to determine statistical significance. For neurocognitive assessment, individual test scores were converted to standard scores (based on age-related means and standard deviations from test standardization norms) with a mean of 100 and a standard deviation of 15. Analysis of variance was used to examine group differences in neurocognitive functioning for the survivors. All statistical analyses were performed using the SPSS software package (version 15.0: SPSS, Chicago, IL, U.S.A.).

## 3. RESULTS

### 3.1 Patient Characteristics at Diagnosis

From April 1985 to April 2007, 34 consecutive patients (19 boys, 15 girls) with a histologically confirmed diagnosis of medulloblastoma were diagnosed and treated at our institution. All were 36 months of age or younger. Median age at diagnosis was 24 months (range: 1–36 months), and 7 patients were less than 12 months old. Median duration of presenting symptoms before diagnosis was 0.8 month (range: 1 week to 6 months).

All patients underwent a surgical procedure, and 5 patients subsequently received postoperative palliation for extensive disease and poor surgical recovery. The patient characteristics, treatment descriptions, and outcomes data that follow are therefore given for the 29 patients who received postoperative therapy with curative intent. Of these 29 patients, 17 underwent gtr or rstr (≥ 95% resection), 10 underwent str, and 2 underwent pr.

In 6 patients (21%), staging investigations (craniospinal axis imaging and csf cytology analysis) were insufficient to provide an accurate metastatic status at diagnosis. Metastasis at diagnosis was found in 15 patients (52%: 4 M1, 4 M2, 6 M3, 1 M2/M3). Histopathologic subtype was desmoplastic in 7 patients (24.1%), anaplastic in 1 patient (3.4%, and classic medulloblastoma in the remaining 21 patients (72.5%).

### 3.2 Chemotherapy

Chemotherapy with curative intent was prescribed in all 29 patients. Table I details the chemotherapy regimens used. Adjuvant chemotherapy alone was used in 21 patients, with the intent to delay or avoid radiation. This chemotherapy-alone strategy was successfully completed in 5 of the 21 patients (24%), who remain in continuous complete remission 133, 98, 65, 51, and 23 months after diagnosis. Myeloablative hdc followed by stem cell rescue (scr) was given to 8 patients: 6 received this therapy as part of consolidation of initial induction therapy, and 2 received it at the time of relapse or for residual disease. Among these 8 patients, 1 died of toxicity, and 4 remain alive at 81, 51, 36, and 23 months post diagnosis. Median time to progression or relapse for the patients treated with adjuvant chemotherapy alone was 6.7 months (range: 0.4–47.7 months).

### 3.3 Radiation Therapy

Radiation in an adjuvant setting was administered in 8 patients, 7 of whom received csi and a focal boost. In 1 patient who had an incomplete resection at diagnosis, intensity-modulated radiotherapy (5400 cGy) was administered to the original tumour volume after hdc and scr. At a median follow-up of 81 months, 6 of the 8 irradiated patients (75%) remain alive.

In 4 patients, salvage radiotherapy was administered at the time of recurrence or progression. Of these 4 patients, 2 received conventional csi, 1 received stereotactic radiation (5400 cGy) to the tumour bed after hdc and scr, and 1 received a reduced dose of csi (1800 cGy), with a posterior fossa boost, for local relapse 1 year after high-dose chemotherapy. Only 1 of these 4 patients patient is a long-term survivor at 108 months from diagnosis.

### 3.4 Survival and PFS

Deaths from their disease occurred in 15 patients, from a secondary malignancy (T-cell lymphoma) in 1 patient, and from treatment-related toxicity in 1 patient. At a median follow-up of 90 months (range: 23–194 months), 12 patients remain alive. Progression or relapse was focal in 7 patients (41%), distant in 3 patients (18%), and combined focal and distant in 7 patients (41%).

The 5-year pfs and os were 35.9% ± 9.8% and 50.2% ± 9.6% respectively. None of the following factors were found to be significant for os and event-free survival: age younger than 12 months, extent of resection, and desmoplastic histology. Although nonsignificant, a trend for a better survival was observed in patients presenting with metastatic disease at diagnosis (5-year os of 64.2% ± 13.2%, Table II). The use of adjuvant conventional csi was the only factor associated with a significantly better 5-year pfs (*p* =0.013) and an os of 72.9% ± 16.5% (*p* = 0.032).

Table III summarizes the characteristics of the surviving patients.

### 3.5 Neurocognitive Evaluation

For the 12 surviving patients, neurocognitive outcome was compared between the 6 patients treated with conventional csi (group A) and the 6 patients treated with chemotherapy alone or with chemotherapy associated with reduced-dose or limited-volume radiation (group B). Group A was considered a historical control group.

Age at diagnosis was significantly different between the two groups (2.33 years for group A vs. 1.55 years for group B, *p* < 0.05). Differences in intelligence, receptive vocabulary, visual–motor skills, academics, and memory were evaluated at a single time (mean of 4.7 years after diagnosis) for all patients. Assessment data for each patient were chosen so that age at testing (6.87 years in group A vs. 6.44 years in group B) and time since diagnosis (4.56 years in group A vs. 4.91 years in group B) did not significantly differ between the two groups (*p* > 0.10).

In 10 patients, at least two neuropsychological assessments had been administered during follow-up, and we used repeated-measures analyses of variance to examine longitudinal outcomes from the first to the second assessment. For this serial analysis, patients in group A were older (6.20 years at first assessment and 9.65 years at second assessment) than those in group B (3.59 years at first assessment and 7.13 years at second assessment). Further, median time from diagnosis to first and second assessment was 3.93 years and 7.38 years respectively for group A, which is longer than the comparable medians of 2.14 years and 5.68 years for group B. However, given the small sample size, group means for age at testing and for times since diagnosis were not significantly different.

**FIGURE 1:**
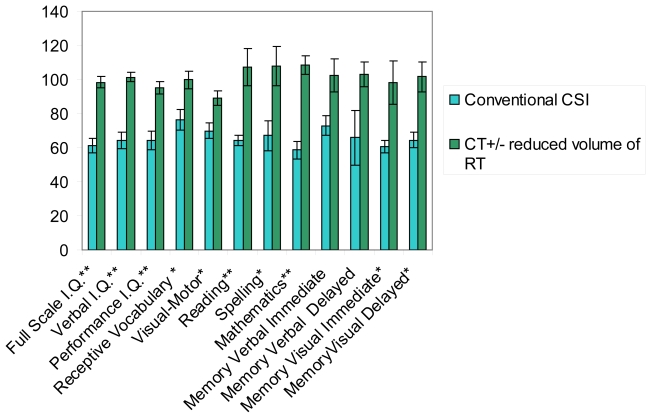
Late effects in survivor patients: neurocognitive outcome at 5 years after diagnosis using the Wechsler Intelligence Scale for Children [full scale intelligence quotient (iq), verbal iq, performance iq], the Receptive Vocabulary test, the Beery Visual–Motor Integration Test, the Wide Range Achievement Test (reading, spelling, mathematics), and the Children Memory Scale (memory verbal and memory visual). * Significantly different at p < 0.05. ** Significantly different at p < 0.01.

For all measures, Figure 1 sets out the means and standard deviations at 4.7 years of follow-up. Compared with patients treated with chemotherapy with or without reduced radiation, patients treated with conventional csi demonstrated significantly lower standard scores for full-scale intelligence quotient (fiq), visual iq (viq), performance iq (piq), receptive vocabulary, visual–motor skills, reading, spelling, mathematics achievement, and visual memory. The difference for verbal memory did not reach statistical significance. In general, patients treated with conventional csi (group A) demonstrated significant deficits in neurocognitive function; those treated with chemotherapy with or without reduced radiation (group B) exhibited cognitive function within the normal range.

**FIGURE 2:**
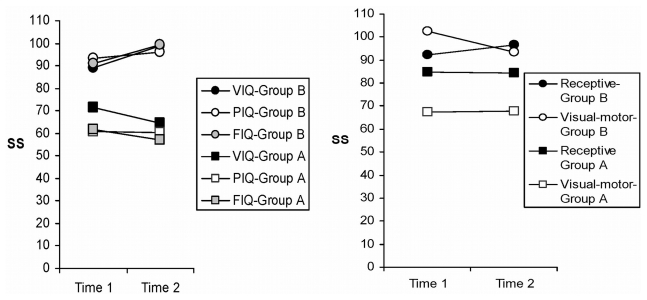
Longitudinal evaluation at two time points after diagnosis, using the Wechsler Intelligence Scale for Children [verbal intelligence quotient (iq), performance iq (piq), full scale iq (fiq)—left panel] and the Receptive Vocabulary test and the Beery Visual–Motor Integration Test (right panel) in surviving patients. Group A (Time 1: 3.98 years; Time 2: 7.38 years) received conventional craniospinal radiation. Group B (Time 1: 2.14 years; Time 2: 5.68 years) received chemotherapy with or without reduced radiotherapy.

A 2×2 (group × assessment) analysis of variance with repeated measures on the last variables was used to examine changes and stability in fiq, viq, piq, receptive vocabulary, and visual–motor skills over time. Compared with the patients in group B, the patients in group A obtained standard scores that were significantly lower for fiq, viq, piq, and visual–motor functioning across both assessments (*p* < 0.02, Figure 2). An interaction between group and time of assessment was significant for fiq, with scores increasing from the first to the second assessment for group B, but remaining stable for group A. A notable exception was receptive vocabulary, which was not different between the groups (*p* > 0.10). Scores fell within the low-average range for group A, which was their highest level of performance.

### 3.6 Endocrine Evaluation

Endocrine status assessment at the last clinical visit was available for 9 of the 12 survivors (in 3 patients, it was still too early after completion of therapy for testing). At a mean follow-up of 108 months post diagnosis (range: 65–194 months), 6 patients had evidence of partial or complete hypopituitarism. Short stature at or below the 10th percentile was found in 5 patients. Of those 5 patients, 2 were on growth hormone (gh) replacement. In 1 case, parents declined gh therapy out of concern that gh might stimulate tumour progression. Precocious puberty developed in 1 patient and was treated with luteinizing hormone–releasing hormone agonist. The 4 patients who did not receive csi are endocrinologically intact. Of those 4 patients, 3 had received chemotherapy alone, and 1 had received high-dose chemotherapy with focal radiation to the tumour bed.

## 4. DISCUSSION

The increased neurotoxic susceptibility of infants with embryonal tumours of the central nervous system has led to the development, since the late 1980s, of innovative strategies to delay or avoid the use of radiation. Here, we reviewed survival and neurocognitive outcomes in infants treated for medulloblastoma at a single institution using these different strategies.

The study may have some inherent limitations because of its retrospective nature and the prolonged time period, encompassing several eras of therapeutic strategies. For example, because the clinical data were accrued over a long time for children at various ages, different versions of the neurocognitive tests were used for different individuals. Further, the sample size for the neurocognitive comparisons is relatively small (although the patient numbers reflect the clinical reality of medulloblastoma). We acknowledge that, had a larger sample size been available, then the neurocognitive effects may have been more pronounced. Despite the foregoing limitations, our study is unique in that it provides comprehensive longitudinal neurocognitive follow-up not evident in the current literature.

The 5-year os rate of 50.2% ± 9.6% in this study is in keeping with previous reports 2,7,10,14–17, with most relapses occurring within the first year after diagnosis (median: 7 months).

Desmoplastic medulloblastoma histology was found in 24% of the patients, a frequency lower than that found in three recent multicentric trials, which demonstrated frequency ranges between 33% and 46% in patients with infant medulloblastoma 8,9,18. This variation in frequency highlights the importance of uniformity in diagnostic criteria for desmoplasia 19, because desmoplastic histology appears to be associated with better outcome and has been proposed as a major element for risk stratification in future infant medulloblastoma protocols 20,21. The reason for the lack of prognostic correlation with desmoplastic histology in our series is unclear, but is likely influenced by the heterogeneity in treatment received by our group of patients.

At diagnosis, 15 patients (52%) had metastatic disease. The actual frequency may be even higher in light of the 20% of patients whose records lacked a complete staging evaluation. The high incidence of disease dissemination is consistent with several other institutional and cooperative group reports 2,22,23. Although nonsignificant, a trend to better outcomes in patients with metastatic disease was observed (*p* = 0.055). This contra-intuitive trend can likely be explained by differences in treatment. The use of adjuvant radiotherapy was significantly associated with a better survival rate (*p* = 0.032), and of the metastatic patients, 66% received radiation as compared with 14% of the M0 patients. Similar trends were reported by Walter *et al.* 2 in a population with infant medulloblastoma treated with chemotherapy and deferred radiotherapy.

We also did not find a significant correlation between extent of surgical resection and survival, most likely because of the small sample size in this series. However, in most recent infant studies, the association of extent of resection with metastatic status at diagnosis has allowed for the identification of risk groups—such as older children—with significant differences in outcome^8^,^9^,^18^,^24^.

In our study, half the patients who received high-dose chemotherapy with scr are long-term survivors. Longer follow-up is necessary, because this finding relates to the more recent cohort. However, these results appear encouraging and should be confirmed in larger prospective clinical trials that include a systematic neurocognitive evaluation of long-term survivors^18^,^25^,^26^.

Although there is growing evidence that omission or delay of radiation in infants allows for preservation of intellectual outcome, neurocognitive evaluations remain critical to appraising the benefits of the new strategies 4,8,9,27. Among the available evaluations, the best approach remains to be determined, not only for assessing the neurocognitive effects of first-line therapy, but also for evaluating the consequences of additional salvage therapy in patients whose initial treatment fails. For instance, in a group of patients treated with salvage therapy (including high-dose chemotherapy and focal radiation), Grill *et al.* 8 reported a mean iq of 72 as compared with 91 in the group of survivors who received conventional chemotherapy only. Similarly, the German ghop group (Gesellschaft fur Padiatrische Onkologie und Hamatologie) reported neurocognitive data on infants treated with methotrexate-based chemotherapy (high-dose systemic, and intraventricular). Although the intellectual outcomes in their series appear to be improved compared with a historical control group treated with radiation, the ghop data also suggested significant decline in intellectual performance for patients who received salvage radiation after methotrexate therapy 9.

With few exceptions, most infant series in the literature are limited in their evaluation of intellectual functioning. Deficits in other neurocognitive domains have been documented in older children with brain tumours treated with cranial radiation. Few attempts have been made to examine broader areas of function in children during infancy or early childhood 12. Our study contributes to this field by assessing, in a longitudinal fashion, multiple areas of neurocognitive functioning in long-term survivors. We present further evidence that strategies avoiding csi in infants are associated with preserved neurocognitive outcome. At a mean interval of 5 years after diagnosis, patients who received csi showed, as expected, evidence of poor neurocognitive outcome across most measures. In this subgroup, standard iq scores were at least 2 standard deviations below the associated normative means. Standard scores for patients treated with chemotherapy alone or with a reduced radiation field were significantly higher, falling within the average range.

A significant observation in our series was that patients treated with less-intensive therapy demonstrated improvement in intellectual functioning over time. Serial assessments are important not only for monitoring functional declines in highly vulnerable patients (such as those treated with cranial radiation), but also for documenting improvement in patients treated with less-intensive therapy. This improvement may reflect functional normalization as the medical condition of these children improved following illness and as they experienced increased learning opportunities that stimulated their behavioural and cognitive development.

Several studies have reported continuous neurocognitive decline after cranial radiation 2,27–30. In our cohort of survivors, although patients treated with conventional csi demonstrated no functional declines across the two assessments, their scores were already at the lowest level by the time of the first assessment, which was performed 3.93 years after diagnosis. Hence, room for further decline was limited. Despite the pervasive neurocognitive deficits in patients treated with adjuvant csi at a young age, some areas of neurocognitive function were preserved. Notably, these patients demonstrated low-average function on a measure of receptive vocabulary. Indices of receptive vocabulary have been found to be relatively robust to the effects of cranial radiation in older children 31. Thus, by examining multiple domains of neurocognitive function, we were able to identify an isolated area of relative strength in patients who typically present with other pervasive deficits. It is therefore important to ensure that all patients have access to comprehensive neuropsychological assessment, because identification of areas of relative strength is important for programming and developing compensatory strategies.

## 6. CONCLUSIONS

This institutional review provides comprehensive longitudinal outcome and neurocognitive data on a population of infants successfully treated for medulloblastoma. Our study provides further indications that alternative therapeutic approaches, such as adjuvant chemotherapy and reduced or no radiation, are associated with encouraging intellectual performances. Efforts are required to better integrate neuropsychological evaluation into the objectives of future infant medulloblastoma protocols.

## Figures and Tables

**TABLE I:** Description of postoperative chemotherapy

Regimen	Patients (*n*)
Conventional chemotherapy	29
Baby pog 1, 2	9
ice	7
8-in-1	3
bbsfop	3
mopp^a^	3
hdc+scr conditioning regimens	8
ccg 99703	3
Busulfan thiotepa^b^	2
Headstart ii	2
Etoposide, carboplatin, thiotepa^b^	1

a One patient received bbsfop after 1 cycle of mopp and 1 prior to high-dose busulfan thiotepa.

b Post conventional chemotherapy.

**TABLE II:** Overall survival according to prognostic factors

Prognostic factor	Patients (*n*)	5-Year os (%)	*p* Value
Age < 12 months at diagnosis	7	28.6±17.1	0.54
Resection > 95%	17	58.2±12.1	0.50
Metastatic disease	15	64.2±13.2	0.055
Desmoplastic histology	7	48.5±11	0.69
Use of adjuvant csi	8	72.9±16.5	0.032

os = overall survival; csi = craniospinal irradiation.

**TABLE III:** Characteristics of the survivors

Pt id	Sex	Age at diagnosis (years)	Pathology	Surgery	Shunt around time of surgery	Metastatic status	Treatment	rt type and dose (cGy)	Age at rt (years)	Follow-up (months)
1	M	1.7	Classic	str	+	M3	ct+rt	csi 3500, boost pf 5000	1.9	194
2	M	2.5	Classic	gtr	+	M3	ct+rt	csi 2880, boost pf 5040	2.6	173
3	F	0.8	Classic	gtr	+	M0	ct alone	–	–	133
4	M	1.9	Classic	str	−	M3	ct+rt	csi 3500	2.6	126
5	F	0.8	Desmoplastic	rstr	−	M0	ct alone	–	–	98
6	M	2.2	Classic	str	+	M2	ct+rt^a^	csi 4140	2.8	108
7	M	3.0	Desmoplastic	gtr	+	M3	ct+rt^a^	csi 3600	3.1	81
8	F	1.4	Classic	gtr	+	M1	hdc+scr+rt	imrt to pf 5400	2.0	81
9	M	2.6	Classic	gtr	+	M0	ct alone	–	–	65
10	M	2.0	Anaplastic	gtr	−	M1	hdc+scr	–	–	23
11	M	2.0	Desmoplastic	gtr	−	M1	hdc+scr	–	–	51
12	F	2.0	Classic	pr	−	M2, M3	hdc+scr+rt	csi 2160, boost pf 5040	3.2	36

a Radiotherapy at time of relapse.

Pt = patient; rt = radiotherapy; M = male; str = subtotal resection; ct = chemotherapy; csi = craniospinal irradiation; pf = posterior fossa; gtr = gross total resection; F = female; rstr = radical subtotal resection; hdc+scr = high-dose chemotherapy and stem cell rescue; imrt = intensity-modulated radiation therapy; pr = partial resection.
